# Modelling the health co-benefits of sustainable diets in the UK, France, Finland, Italy and Sweden

**DOI:** 10.1038/s41430-019-0401-5

**Published:** 2019-02-12

**Authors:** Linda J. Cobiac, Peter Scarborough

**Affiliations:** Centre on Population Approaches for Non-Communicable Disease Prevention, Big Data Institute, Nuffield Department of Population Health, University of Oxford, Oxford, UK

**Keywords:** Risk factors, Cardiovascular diseases, Cancer

## Abstract

**Background/Objectives:**

It is not known if diets lower in greenhouse gas (GHG) emissions are also healthier. We evaluated the population health implications of changing to more sustainable diets in the UK, France, Finland, Italy and Sweden.

**Subjects/Methods:**

We developed a life table model to simulate mortality and morbidity from diet-related diseases over the lifetime of the current population. Populating the model with locally available data for each country, we simulated the impact of country-specific dietary scenarios that had been optimised to meet dietary recommendations and reduce GHG emissions. Outcome measures included a change in disease-specific deaths, life expectancy and disability-adjusted life years (DALYs).

**Results:**

Diets that meet nutritional recommendations lead to substantial improvements in population health, ranging from 0.19 (95% uncertainty interval: 0.18–0.21) DALYs per person in Italy up to 0.89 (0.80–0.98) DALYs per person in Finland. Simultaneously reducing GHG emissions does not reduce the size of this impact, and in some cases produces additional health benefits. If sustainable diets can be maintained throughout adulthood, life expectancy would increase by between 2.3 (1.6–3.2) and 6.8 (5.5–8.5) months by country. However, results are sensitive to assumptions about how quickly changes in diet can influence disease, and future trends in disease.

**Conclusions:**

Modelling the health impact of diets that are both nutritional and low in GHG emissions shows the potential for significant co-benefits in health and sustainability from dietary changes. Future work is needed to find effective interventions to deliver healthy sustainable diets.

## Introduction

In a world where global temperatures are rising and the burden of dietary-related disease is high, the global food system faces major challenges to provide healthy, sustainable foods for all. Poor diet is a leading risk for non-communicable diseases. In 2015, dietary risks, including high red and processed meat intake, high sodium intake and low fruit and vegetable intake, were responsible for 12% and 9% of the global disease burden in men and women, respectively, more than many other lifestyle-related risks such as smoking, high blood pressure and obesity [1].

In a previous review [2], we found highly inconsistent results regarding links between diets with reduced greenhouse gas (GHG) emissions and reduced content of nutrients that should be limited in a healthy diet (salt, saturated fat and sugar). In the cases of salt and saturated fat, the majority of dietary patterns found a reduction in levels of these nutrients in diets with reduced GHG emissions, but the majority of dietary patterns that reported sugar intake showed increased sugar in lower-GHG emission diets. There was also an inconsistent relationship between reduced GHG emissions and health outcomes, with no clear relationship for total mortality and cancer, whereas low-GHG emission diets were estimated to have lower cardiovascular disease (CVD) risk in four out of five modelled diets. However, we did find that decreased micronutrient content of diets was more frequently associated with reduced GHG emissions than increases in micronutrient content, with far more cases in which lower GHG emission diets were associated with decreases in key micronutrients (*n* = 129), than with increases in micronutrients (*n* = 29).

In an accompanying study by Vieux et al. [3], linear programming was used to identify optimal dietary scenarios for the UK, France, Finland, Italy and Sweden, that meet all dietary recommendations, including micronutrient needs, while also reducing GHG emissions. The optimisation study drew on a database of food GHG emissions [4], which had been derived for the European region using comparable life cycle assessment (LCA) methods. In the dietary optimisation study, Vieux et al. [3] evaluated a series of dietary scenarios for each country, which would reduce GHG emissions compared with current diets, in increasing 10% increments up to maximum achievable reductions. In this study, we evaluate the long-term health implications of these dietary scenarios for the populations of the UK, France, Finland, Italy and Sweden.

## Methods

To estimate the health impact of achieving sustainable dietary scenarios in the UK, France, Finland, Italy and Sweden, we built a life table model [5] that could be applied to each of the countries individually on the basis of locally available health data. We modelled the difference between a business-as-usual (BAU) scenario, where disease mortality rates are projected across the life course of the population following recent trends, and intervention scenarios based on the changes in diet determined by optimisation models specified for each country and designed to minimise the difference between scenario diets and currently achieved diets (described by Vieux et al. [3]). For each country, the intervention scenarios included a diet that meets nutrition recommendations, a diet that meets nutrition recommendations but does not increase GHG emissions, a series of diets that meet nutrition recommendations and reduce GHG emissions in increasing 10% increments, and a diet in which GHG emissions are minimised. For each of the dietary scenarios, estimates of GHG emissions by food group were provided by a bespoke LCA [4]. Appendix 1 in the Supplementary Material shows the impact of the diet scenarios on a range of non-communicable disease risks, including intake of fruits and vegetables, red and processed meats, fibre, sodium, fats (total, saturated, monounsaturated, polyunsaturated) and dietary cholesterol.

The life table model determines change in life expectancy, deaths from disease, and disability-adjusted life years (DALYs), with impact on deaths and DALYs calculated across the entire life course of the population or across an arbitrary time horizon (e.g. 10 years). Following the precedent of the Global Burden of Disease (GBD) Study [1], we did not apply a discount rate to future health outcomes. The DALY measure reflects the combined effect on quantity (i.e. mortality) and quality (i.e. morbidity) of life. In our model, the DALY was calculated from the number of years of life that are lived by the population, with adjustment to reflect time spent in ill-health (i.e. with ‘disability’ from diseases). Disability rates were estimated from rates of prevalent years lived with disability (YLD) in the GBD Study. These YLDs have been estimated from age-, sex- and country-specific disease prevalence and disease-specific disability weights, which were derived from a large international survey of health loss associated with 220 unique disease states.

Rates of disease-specific mortality and disability were downloaded for each country from the GBD database [6]. For our BAU scenarios, we used data for the year 2010. We estimated future annual average BAU trends in mortality and disability rates from trends in GBD estimates since 1990. Population numbers and all-cause mortality for each country were downloaded from the Human Mortality Database [7].

Diseases are influenced by diet directly (e.g. processed meat intake and colorectal cancer) and indirectly via intermediate variables (e.g. blood pressure and blood cholesterol). We included dietary risk factors in the model where there was evidence of significant effects (*p* < 0.05) in meta-analyses of studies that have quantified diet–disease associations. The diseases influenced by changes in intake of foods (vegetables, fruits, red and processed meats) and nutrients (fibre, sodium and fats) in the sustainable diet modelling, included coronary heart disease (CHD), stroke, type 2 diabetes, breast cancer, colorectal cancer, lung cancer and stomach cancer (Table 1). While whole grains and dairy intake are significantly associated with disease outcomes, they were excluded from these sustainable diet analyses because there was insufficient nutrition survey data to calculate whole grain and dairy effects for all countries from the results of the linear programming analyses (described in [3]). Since the sustainable diet scenarios were derived assuming no change in energy intake, we also did not model an effect on body mass index and related diseases in these analyses.

**Table 1:** Dietary and related metabolic risk factors, population exposure to risks and disease outcomes

Risk factor	Exposure parameters	Outcomes
Fruit intake	Mean (SD) g/day for consumers and % consuming <1 fruit portion daily TMRED: 300 (30) g/day [27]	CHD [28]; Stroke [29]; Lung cancer [30]
Vegetable intake	Mean (SD) g/day for consumers and % consuming <1 vegetable portion daily TMRED: 400 (30) g/day [27]	CHD [28]; Lung cancer [30]
Fibre intake	Mean (SD) g/day TMRED: 30 (3) g/day [27]	Breast cancer [31]; Colorectal cancer [32]; Stomach cancer [33]
Fibre intake (cereal only)	Mean (SD) g/day	CHD [34]
Red meat intake	Mean (SD) g/day TMRED: 100 (10) g/week [27]	Colorectal cancer [35]; Stomach cancer [36]; Type 2 diabetes [37]
Processed meat intake	Mean (SD) g/day TMRED: 0 g/day [27]	Colorectal cancer [35]; Type 2 diabetes [37]
Sodium	mmol/24 h	Blood pressure [38]
Total fat Saturated fat Monounsaturated fat Polyunsaturated fat Dietary cholesterol	% of total energy % of total energy % of total energy % of total energy mg/day	Total cholesterol [39]
Blood pressure	Mean (SD) mmHg TMRED: 115 (6) mmHg [40]	CHD [41]; Stroke [41]
Total cholesterol	Mean (SD) mmol/L TMRED: 3.8 (0.6) mmol/L [40]	CHD [42]; Stroke [42]

*CHD* coronary heart disease, *SD* standard deviation, *TMRED* theoretical minimum risk exposure distribution

Baseline dietary intake for fruits, vegetables, fibre, red meats and processed meats were derived from country-specific nutrition surveys (described by Vieux et al. [3]). Baseline systolic blood pressure and total cholesterol levels were derived from risk factor measurement data for the UK [8], France [9], Finland [10], Italy [11, 12] and Sweden [13].

To quantify the impact of the dietary changes on health outcomes we calculated population impact fractions (PIFs), and applied these to the mortality and disability rates that are used in the BAU scenarios. The PIF estimates the percentage change in disease mortality or disability rates that would be expected given a change of distribution of a risk factor in a population, and is given by the following formula:

$$\mathrm{PIF} = \frac{{\mathop {\int}\limits_a^b {p(x)\mathrm{RR}(x)dx - \mathop {\int}\limits_a^b {p{\prime}(x)\mathrm{RR}(x)dx} } }}{{\mathop {\int}\limits_a^b {p(x)\mathrm{RR}(x)dx} }}$$

where *p*(*x*) is the current prevalence distribution of a risk factor; *p*′(*x*) is the prevalence distribution of the risk factor after the diet is changed; RR(*x*) is the distribution function for the relative risk of disease; and *a* and *b* are the lower and upper bounds of the integration (representing upper and lower thresholds of dietary parameters beyond which no evidence is available for further risk reduction).

Where diseases are affected by more than one risk factor, we combined PIFs multiplicatively. To prevent double-counting of effects from the change in multiple dietary parameters (e.g. CHD effects from changes in fruit, vegetable and fibre intake), we reduced excess risks by applying adjustment factors derived for GBD analyses [14].

We modelled all relative risks exponentially, assuming theoretical minimum risk exposure levels as defined in GBD and comparative risk assessment studies (Table 1). We applied a lag in the effect of dietary changes on disease of 5 years for CVDs and diabetes, and 20 years for all cancers. This lag was implemented by applying the average of the PIF over the preceding lag period (e.g. 5 years for CHD).

We examined the sensitivity of modelling results to the lag assumptions by evaluating a scenario where all lags were removed and a scenario in where lags were assumed to be longer (10 years for CVDs and diabetes and 30 years for all cancers). We also examined the sensitivity of modelling results to removal of the BAU trends in disease.

Finally, we evaluated uncertainty in the model outputs using Monte Carlo analysis. All uncertainty in relative risks and theoretical minimum risk exposure levels was captured using distributions described in Table 1 and the accompanying literature. The number of model iterations was based on achieving results that were stable when rounded to two significant figures (approximately 2000 iterations).

## Results

**Fig. 1:**
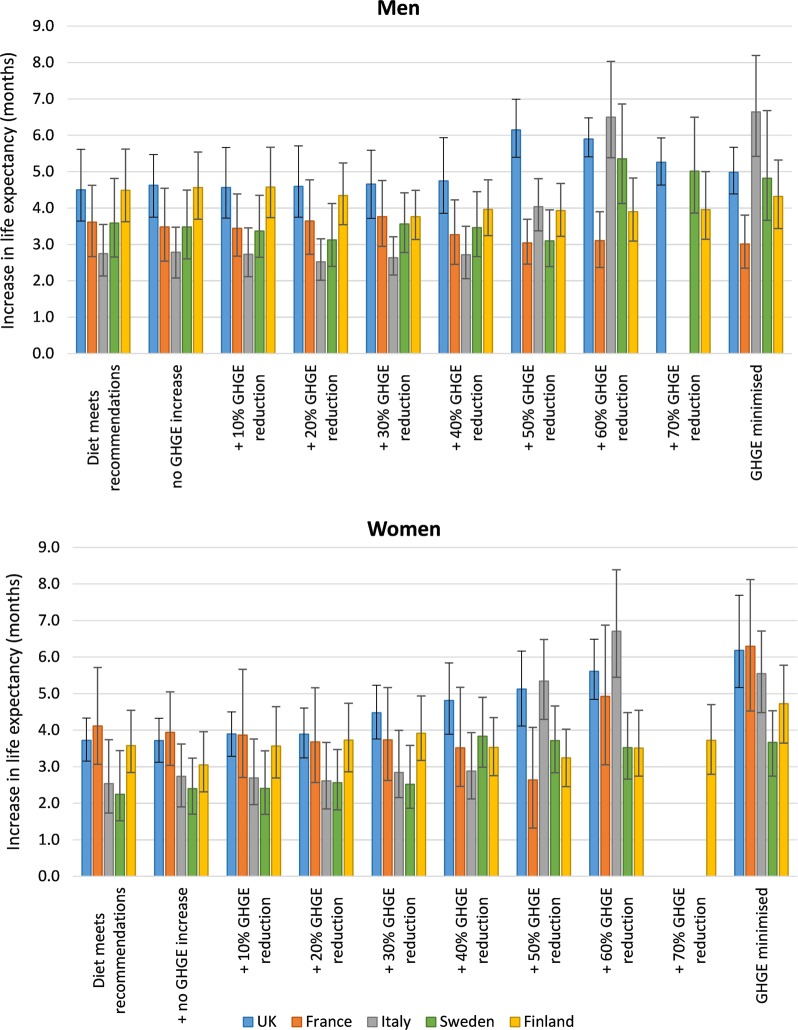
Increase in life expectancy with the dietary and greenhouse gas emission (GHGE) reduction scenarios

Achieving a diet that meets nutrition recommendations leads to substantial population health gains (Table 2). The *per person* (which allows for differences in population size to produce comparable country-level results) health gains is 0.55 (95% uncertainty interval: 0.51–0.59) DALYs in the UK, 0.30 (0.27–0.32) DALYs in France, 0.19 (0.18–0.21) DALYs in Italy, 0.28 (0.26–0.31) DALYs in Sweden and 0.89 (0.80–0.98) DALYs in Finland. Even more health is gained with diets that are additionally designed to reduce GHG emissions. The additional health gain ranges from 7% (0–21%) additional DALYs (for French men) up to 205% (164–244%) additional DALYs (for Italian women). The modelling shows that if diets can be maintained throughout adult life, young people entering adulthood might expect to live between 2.3 (1.6–3.2) and 6.8 (5.5–8.5) months longer on average (Fig. 1).

**Table 2:** DALYs averted (millions) with the dietary scenarios

	UK	France	Italy	Sweden	Finland
**Men**					
Diet meets nutrition recommendations	15 (13–17)	7.0 (6.3–7.7)	4.7 (4.3–5.1)	1.4 (1.3–1.5)	2.3 (2.1–2.6)
+no GHGE increase	15 (14–17)	6.8 (6.2–7.5)	4.7 (4.3–5.1)	1.3 (1.2–1.4)	2.3 (2.1–2.6)
+10% GHGE reduction	15 (14–17)	6.7 (6.2–7.5)	4.6 (4.3–5.0)	1.3 (1.2–1.4)	2.3 (2.1–2.6)
+20% GHGE reduction	15 (14–17)	7.0 (6.3–7.8)	4.2 (3.9–4.5)	1.2 (1.1–1.3)	2.3 (2.0–2.5)
+30% GHGE reduction	15 (14–17)	7.3 (6.5–8.0)	4.5 (4.2–4.8)	1.3 (1.2–1.4)	2.2 (1.9–2.4)
+40% GHGE reduction	16 (14–18)	6.4 (5.8–7.1)	4.7 (4.3–5.0)	1.3 (1.2–1.4)	2.5 (2.2–2.8)
+50% GHGE reduction	21 (19–23)	5.9 (5.4–6.6)	7.4 (7.0–7.9)	1.1 (1.0–1.3)	2.5 (2.2–2.8)
+60% GHGE reduction	20 (18–23)	5.8 (5.3–6.4)	12.4 (11.7–13.2)	2.0 (1.9–2.2)	2.5 (2.2–2.8)
+70% GHGE reduction	20 (17–22)	–	–	1.8 (1.7–2.0)	2.6 (2.3–2.9)
GHGE minimised	19 (17–22)	5.5 (5.0–6.1)	12.6 (11.7–13.4)	1.8 (1.7–2.0)	2.5 (2.2– 2.8)
**Women**					
Diet meets nutrition recommendations	13 (11–14)	7.9 (7.2–8.7)	4.9 (4.5–5.4)	0.8 (0.7–0.8)	1.6 (1.3–1.8)
+no GHGE increase	13 (11–14)	7.7 (7.1–8.5)	5.1 (4.7–5.6)	0.8 (0.7–0.9)	1.5 (1.3–1.7)
+10% GHGE reduction	13 (12–14)	6.5 (5.8–7.2)	4.9 (4.5–5.5)	0.8 (0.7–0.9)	1.7 (1.4–2.0)
+20% GHGE reduction	13 (12–14)	6.2 (5.5–7.0)	4.8 (4.4–5.4)	0.8 (0.8–0.9)	1.7 (1.5–2.1)
+30% GHGE reduction	16 (14–18)	6.3 (5.6–7.0)	5.5 (5.1–6.0)	0.9 (0.8–0.9)	1.8 (1.5–2.1)
+40% GHGE reduction	17 (16–19)	5.9 (5.2–6.6)	5.7 (5.2–6.2)	1.5 (1.4–1.6)	1.9 (1.6–2.3)
+50% GHGE reduction	18 (16–20)	4.6 (4.0–5.3)	11.3 (10.5–12.2)	1.5 (1.4–1.6)	1.8 (1.5–2.2)
+60% GHGE reduction	19 (17–21)	11.4 (9.9–13.1)	14.7 (13.5–16.1)	1.4 (1.3–1.6)	2.0 (1.7–2.3)
+70% GHGE reduction	–	–	–	–	2.1 (1.8–2.4)
GHGE minimised	20 (18–22)	15.1 (13.3–17.4)	12.5 (11.5–13.7)	1.5 (1.4–1.6)	2.3 (1.9–2.7)

Values are mean and 95% uncertainty intervals, in millions. Where values are missing, no solution could be found in the linear programming

Table 3 shows the dietary changes for the diet scenarios that lead to maximum health outcomes in the health modelling (full scenario results are presented, by country, in Appendix 1 of the Supplementary Material). The diet scenarios that lead to maximum health outcomes are typically associated with large reductions in consumption of red and processed meats, salt and fats, and increases in fibre. These diet scenarios are also associated with net increases in fruits and vegetables, with the exception of diets for Sweden, where the increases in fibre are chiefly derived from increases in grain-based carbohydrate foods rather than increases in fruits and vegetables.

**Table 3:**
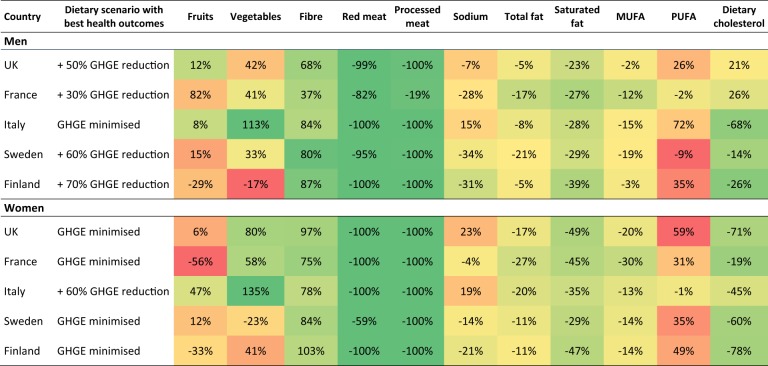
Dietary changes for the scenarios that achieve the largest lifetime health gain in DALYs

There is no change in total energy intake in the dietary scenarios

The colour gradient is from red (largest *decrease* in fruits, vegetables and fibre; largest *increase* in all other dietary variables) to green (largest *increase* in fruits, vegetables and fibre; largest *decrease* in all other dietary variables)

*MUFA* monounsaturated fatty acids, *PUFA* polyunsaturated fatty acids

**Fig. 2:**
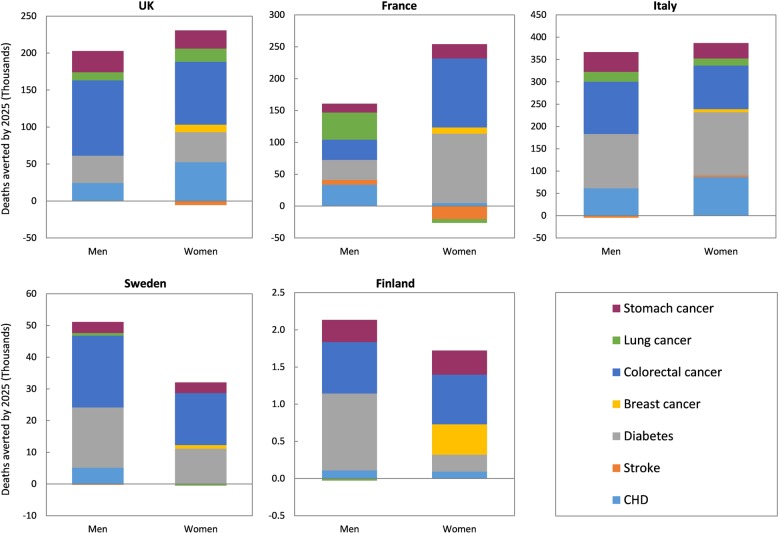
Deaths averted by 2025 for the dietary scenarios that achieve the largest lifetime health gain in disability-adjusted life years (DALYs)

Figure 2 shows the number of disease deaths that would be averted in each country by 2025, for the dietary scenarios that lead to maximum health outcomes in the health modelling (full scenario results are presented, by country, in Appendix 2 of the Supplementary Material). A large proportion of the health benefits are from reductions in cases of colorectal cancer and diabetes. This is primarily due to the big reductions in red and processed meat intake that occur in the diet scenarios with the largest health benefits. Reductions in CHD, stomach cancer, lung cancer, breast cancer and stroke, also contribute to the modelled health gain. There are small increases in deaths from stroke, lung cancer and CHD, in some countries (e.g. women in France), which are primarily mediated by reductions in fruit intake seen in these scenarios. However, the magnitude of the harm is in all scenarios strongly outweighed by the potential health benefits.

The results are somewhat sensitive to time lags in dietary effects on disease and to background trends in disease rates. The sensitivity of the dietary scenarios that are associated with the largest health outcomes in each country (e.g. a 50% GHG emission reduction for men and minimisation of GHG emissions for women, in the UK) are shown in Table 4 (results for all other scenarios are presented in Appendix 3 of the Supplementary Material). There is no change in the specific scenarios associated with maximum health outcomes when time lags are removed or increased, or when background disease trends are removed, however, the magnitude of health benefits associated with these scenarios does vary. The size of the net health gain is between 6% and 16% greater if we assume an immediate effect of dietary changes on disease outcomes (i.e. time lags are removed), but between 6% and 12% less if time lags are actually longer than we have assumed in our base case analyses (10 years vs. 5 years for CVDs and diabetes, and 30 years vs. 20 years for cancers).

**Table 4:** Sensitivity of the health modelling results to changes in the lags in dietary effects on disease and the disease trends, shown for the scenarios that achieve the largest lifetime health gain in DALYs

Country	Dietary scenario with best health outcomes	Millions of DALYs averted (% change^a^)			
		Base case	No lags	Longer lags	No trends
**Men**					
UK	+ 50% GHGE reduction	21	22 (+7%)	19 (−7%)	19 (−9%)
France	+ 30% GHGE reduction	7.3	8.1 (+11%)	6.5 (−10%)	8.7 (+20%)
Italy	GHGE minimised	13	14 (+13%)	11 (−11%)	18 (+43%)
Sweden	+ 60% GHGE reduction	2	2.2 (+10%)	1.8 (−10%)	2.2 (+12%)
Finland	+ 70% GHGE reduction	2.5	2.7 (+7%)	2.3 (−7%)	2.5 (-0.1%)
**Women**					
UK	GHGE minimised	20	21 (+7%)	18 (−7%)	20 (+3%)
France	GHGE minimised	15	16 (+8%)	14 (−8%)	16 (+4%)
Italy	+ 60% GHGE reduction	15	16 (+11%)	13 (−9%)	20 (+38%)
Sweden	GHGE minimised	1.5	1.6 (+9%)	1.4 (−9%)	1.7 (+11%)
Finland	GHGE minimised	2.3	2.5 (+8%)	2.1 (−8%)	2.5 (+8%)

^a^Percent change in comparison to DALYs averted in base case scenarios

When we ignore the effects of background trends in disease in our analyses, there is a median increase in health gain of 12% (i.e. the application of disease trends in our base case scenarios has the overall effect of lessening the future disease burden that can be averted by the dietary scenarios). However, this does vary widely by country: the median increase is larger for Italy (+45%), France (+17%) and Sweden (+17%), smaller in Finland (+3%) and negative in the UK (−6%). The negative effect in the UK is primarily for men (range is between −18% and −6% for men and between −1% and 3% for women). The differences between countries and genders are due to differences in magnitude and direction of background disease trends (e.g. stemming from different stages in progression of tobacco and obesity epidemics), combined with differences in dietary changes between scenarios (e.g. trends in diabetes and colorectal cancer will have more influence in scenarios with large changes in processed meats, whereas trends in CHD and stroke will have more influence in scenarios with large changes in sodium).

## Discussion

Modelling the health impact of diets that are both nutritional and low in GHG emissions shows the potential for significant co-benefits in health and sustainability from changes in diet. Switching to a diet that meets nutrition recommendations could lead to substantial reductions in death and disability from diabetes, cancers and CVDs for populations of the UK, France, Italy, Sweden and Finland. Simultaneously reducing GHG emissions from the diet will not reduce the size of this impact, and in some cases there could be *added* health benefits. However, it would require substantial changes to current diets in all countries. The food system contributes a quarter of the global GHG emissions [15], and given the increasing global population substantial reductions in emissions from the food system cannot be achieved by technological improvements alone [16]. Therefore, these dietary scenarios, if achieved, could make a substantial contribution to limiting damaging climate change and could therefore make further contributions to public health via reduced risk of flooding, heat exposure and changes in infectious disease vectors [17].

Although our conclusion that there are significant health and sustainability co-benefits from changing diet did not alter with changes in our modelling assumptions, the magnitude of the potential health gain does depend on how long we assume it takes for diet to have an effect on disease outcomes and whether we assume current trends in disease are sustained in the future. It should also be noted that although we have made every effort to avoid double counting in the risk factor–disease relationships included in the model, it has not been possible to remove all possibility of double counting due to ambiguities about the exact causal pathways between dietary risk factors and disease outcomes. Since it is necessary to source risk parameters from multiple systematic reviews, it is not possible to obtain values that have been mutually adjusted for each other. The relative risk parameters we have included in the model are from meta-analyses of observational studies (mostly prospective cohort studies). While these observational studies adjust for potential confounding (e.g. by age, sex and smoking), residual confounding may still occur if explanatory variables are missing or poorly measured. Additionally, similar to other modelling exercises that have estimated the impact of diet on health [18–20] and the environment [21], we have calculated the impact of separate dietary variables and then combined them. An alternative would be to model the impact of more holistic changes in diet, for example towards a Mediterranean dietary pattern which has shown to be beneficial to health [22]. The impact of holistic changes in diet may be more or less than the combination of changes of individual dietary elements. This study focused on quality rather than quantity of diet. Given that around 5–7% of disease burden has been attributed to high body mass in these European countries [23], there is potential to achieve further health benefits by also reducing energy intake. A reduction in food intake might also lead to a reduction in GHG emissions.

It is important to note that the Vieux et al. [3] optimisation of the dietary scenarios that we modelled in this study, relied only on GHG emissions as a measure of environmental impact. Incorporating other dimensions of impact, such as land use, water and biodiversity, may influence the characteristics of the optimal diets, particularly if these impacts are not positively correlated with GHG emissions. Furthermore, the measurement of GHG emissions associated with food items, which are relied on in the optimisation process, is far from an exact science. Hartikainen et al. [4] developed a database of food GHG emissions from studies that had used comparable LCA methods for evaluating GHG emissions associated with foods in the European region, but there was considerable uncertainty in derived values due to variability in the availability of evidence across different food groups and variability in measures of GHG emissions for specific food items. Data were also insufficient overall to determine food GHG emissions specific to the five countries in the study. For all of these reasons, it is likely that there is greater uncertainty in the modelled health impacts of the sustainable dietary scenarios in our study than is reflected in the 95% uncertainty intervals.

Our modelling results are broadly consistent with the results of previous studies, although direct comparison is not possible due to differences in modelling structures and/or modelled populations. In an analysis of the EPIC-NL prospective cohort study, where the cohort was divided into groups based on GHG emissions of the baseline diet, Biesbroek et al. [24] found no association between GHG emissions and all-cause mortality (hazard ratio for highest quartile vs. lowest quartile: 1.00 (0.86–1.17)). However, using food group-specific hazard ratios drawn from the same cohort to estimate the health impact of a modelled diet aimed at reducing GHG emissions, the researchers found that replacing 35 g per day of meat with vegetables, fruits, nuts, seeds and grains, could increase survival rates by 6–19%. This is further supported by the results of a UK study by Scarborough et al. [20], who modelled the health implications of three low-GHG emissions dietary scenarios derived by the Committee on Climate Change [25]: (1) a 50% reduction in meat and dairy, replaced by fruit, vegetables and cereals; (2) a 75% reduction in beef and sheep, replaced by pigs and poultry; and (3) a 50% reduction in pigs and poultry, replaced by fruit, vegetables and cereals. The researchers found that all scenarios led to a reduction in GHG emissions and an improvement in health (deaths averted or delayed), with the biggest environmental and health gains from the scenario that reduced both meat and dairy (scenario 1).

Like these previous studies, the Vieux et al. [3] optimisation modelling that was used to determine the scenarios we have evaluated for the UK, France, Italy, Sweden and Finland, found that reducing GHG emissions of the diet whilst maintaining the nutritional quality of the diet and deviating as little as possible from the current consumed diet will inevitably result in large reductions in red meat and processed meat consumption. This has many implications both in terms of changing food consumption and food production behaviours. With regard to changing consumer behaviour, it is recognised that meat consumption has social connotations that go far beyond its role in a nutritional diet and therefore developing interventions designed to modify meat consumption patterns will be challenging. It is noted, however, that long-term changes in meat consumption patterns measured by the UK Living Cost and Food Survey [26], for example, demonstrate that large population shifts in dietary behaviour are possible. The scale of dietary change required for the higher levels of GHG reduction in the dietary scenarios used for these analyses are largely unprecedented in recent history, however dietary changes for lower GHG reduction (up to 20% GHG emission reduction) are more modest and the health modelling shows that they could be associated with large health benefits. Further research is needed to find interventions that are effective in encouraging consumption and production of lower GHG emission foods.

## Conclusions

The global food system faces major challenges in the near future in order to provide healthy, sustainable diets for all. In Europe, it is not clear whether shifts towards low GHG emissions dietary consumption patterns will necessarily result in healthier diets, but they are likely to result in diets with worse micronutrient quality unless appropriate interventions are employed to improve the nutritional quality of diets. Modelling the health impact of diets that are both nutritional and low in GHG emissions shows the potential for significant co-benefits in health and sustainability from substantial changes in the population diet. Future work is needed to find effective interventions to encourage the production and consumption of healthy and sustainable foods.

## Supplementary information

Appendix 1

Appendix 2

Appendix 3
