# Supplementary material for: Modelling the health co-benefits of sustainable diets in the UK, France, Finland, Italy and Sweden
Source: Eur J Clin Nutr. 2019 Feb 12;73(4):624–33. doi: 10.1038/s41430-019-0401-5 (PMC6484724; doi:10.1038/s41430-019-0401-5)
Supplement: Supplementary file 1 — Appendix 1 [file 41430_2019_401_MOESM1_ESM.docx]

**Appendix 1: Dietary scenarios for the United Kingdom, France, Finland, Italy and Sweden**

Table 1 Modelled dietary changes from the linear programming, United Kingdom

|  | Fruits (g/day) | Vegetables (g/day) | Processed meat (g/day) | Red or livestock meat (g/day) | Fibre (g/day) | Sodium (g/day) | Total fat (% total energy) | Saturated fat (% total energy) | MUFA (% total energy) | PUFA (% total energy) | Dietary cholesterol (mg/d) |
| --- | --- | --- | --- | --- | --- | --- | --- | --- | --- | --- | --- |
| Men - baseline mean (SD) | 200 (239) | 219 (145) | 56 (51) | 66 (49) | 15 (6) | 2.6 (1.1) | 33% (7%) | 12% (4%) | 12% (3%) | 6% (1%) | 273 (167) |
| Diet meets recommendations | -17% | 52% | -99% | 40% | 67% | -7% | -6% | -23% | -1% | 18% | -19% |
| + no GHGE increase | -11% | 61% | -99% | -9% | 67% | -7% | 0% | -16% | 3% | 23% | 24% |
| + 10% GHGE reduction | -12% | 62% | -99% | -26% | 67% | -7% | 0% | -16% | 2% | 25% | 36% |
| + 20% GHGE reduction | -15% | 63% | -99% | -44% | 67% | -7% | 0% | -16% | 1% | 26% | 36% |
| + 30% GHGE reduction | -18% | 64% | -99% | -61% | 67% | -7% | 0% | -16% | 1% | 26% | 33% |
| + 40% GHGE reduction | -12% | 60% | -99% | -80% | 67% | -7% | 0% | -16% | 1% | 27% | 31% |
| + 50% GHGE reduction | 12% | 42% | -99% | -100% | 68% | -7% | -5% | -23% | -2% | 26% | 21% |
| + 60% GHGE reduction | 19% | -3% | -99% | -100% | 68% | -7% | -4% | -34% | 7% | 38% | -47% |
| + 70% GHGE reduction | -42% | -7% | -100% | -100% | 67% | -7% | -3% | -35% | 12% | 41% | -78% |
| *GHGE minimised* | *-42%* | *-17%* | *-100%* | *-100%* | *67%* | *-7%* | *2%* | *-23%* | *8%* | *43%* | *-74%* |
| Women - baseline mean (SD) | 187 (159) | 202 (118) | 38 (26) | 55 (32) | 13 (5) | 1.9 (0.7) | 33% (6%) | 12% (3%) | 12% (2%) | 6% (2%) | 206 (112) |
| Diet meets recommendations | -3% | 18% | -98% | -63% | 97% | 23% | -7% | -33% | -1% | 31% | -40% |
| + no GHGE increase | -3% | 18% | -98% | -63% | 97% | 23% | -7% | -33% | -1% | 31% | -40% |
| + 10% GHGE reduction | 5% | 20% | -98% | -67% | 97% | 23% | -8% | -33% | -2% | 32% | -41% |
| + 20% GHGE reduction | -9% | 31% | -98% | -80% | 97% | 23% | -8% | -34% | -3% | 33% | -42% |
| + 30% GHGE reduction | -25% | 36% | -98% | -97% | 97% | 23% | -8% | -34% | -4% | 33% | -33% |
| + 40% GHGE reduction | -49% | 57% | -98% | -100% | 97% | 23% | -10% | -36% | -5% | 33% | -49% |
| + 50% GHGE reduction | -45% | 72% | -98% | -100% | 97% | 23% | -11% | -37% | -5% | 32% | -53% |
| + 60% GHGE reduction | 7% | 41% | -100% | -100% | 97% | 23% | -14% | -44% | -7% | 31% | -67% |
| *GHGE minimised* | *6%* | *80%* | *-100%* | *-100%* | *97%* | *23%* | *-17%* | *-49%* | *-20%* | *59%* | *-71%* |

Table 2 Modelled dietary changes from the linear programming, France

|  | Fruits (g/day) | Vegetables (g/day) | Processed meat (g/day) | Red or livestock meat (g/day) | Fibre (g/day) | Sodium (g/day) | Total fat (% total energy) | Saturated fat (% total energy) | MUFA (% total energy) | PUFA (% total energy) | Dietary cholesterol (mg/d) |
| --- | --- | --- | --- | --- | --- | --- | --- | --- | --- | --- | --- |
| Men - baseline mean (SD) | 212 (172) | 158 (100) | 48 (33) | 73 (41) | 18 (7) | 3.3 (1.2) | 36% (5%) | 14% (3%) | 13% (3%) | 5% (1%) | 354 (127) |
| Diet meets recommendations | 67% | 53% | -82% | 51% | 37% | -28% | -17% | -27% | -14% | -4% | -10% |
| + no GHGE increase | 65% | 44% | -82% | 42% | 37% | -28% | -18% | -27% | -13% | -6% | -2% |
| + 10% GHGE reduction | 75% | 41% | -82% | 25% | 37% | -28% | -16% | -27% | -10% | 0% | 22% |
| + 20% GHGE reduction | 90% | 41% | -82% | 2% | 37% | -28% | -17% | -27% | -10% | -1% | 29% |
| + 30% GHGE reduction | 82% | 41% | -82% | -19% | 37% | -28% | -17% | -27% | -12% | -2% | 26% |
| + 40% GHGE reduction | 22% | 41% | -82% | -23% | 37% | -28% | -17% | -27% | -12% | 1% | 23% |
| + 50% GHGE reduction | 23% | 14% | -82% | -23% | 53% | -28% | -12% | -27% | -7% | 21% | 15% |
| + 60% GHGE reduction | -9% | 30% | -70% | -84% | 62% | -28% | -13% | -27% | -12% | 30% | 3% |
| *GHGE minimised* | *-11%* | *32%* | *-62%* | *-84%* | *64%* | *-28%* | *-11%* | *-27%* | *-13%* | *41%* | *5%* |
| Women - baseline mean (SD) | 219 (139) | 155 (81) | 30% | 52 (22) | 15 (5) | 2.5 (0.8) | 38% (5%) | 14% (2%) | 13% (2%) | 6% (1%) | 276 (86) |
| Diet meets recommendations | 68% | 35% | -89% | 11% | 62% | -4% | -28% | -30% | -30% | -26% | -16% |
| + no GHGE increase | 66% | 23% | -89% | -16% | 62% | -4% | -27% | -30% | -28% | -21% | -8% |
| + 10% GHGE reduction | 56% | 63% | -53% | -27% | 62% | -4% | -26% | -30% | -27% | -20% | 12% |
| + 20% GHGE reduction | 56% | 58% | -53% | -7% | 62% | -4% | -26% | -30% | -26% | -19% | 15% |
| + 30% GHGE reduction | 29% | 77% | -53% | -20% | 62% | -4% | -25% | -30% | -26% | -18% | 13% |
| + 40% GHGE reduction | 14% | 78% | -45% | -29% | 62% | -4% | -28% | -35% | -28% | -18% | 9% |
| + 50% GHGE reduction | -27% | 79% | -53% | -29% | 62% | -4% | -26% | -39% | -24% | 3% | -1% |
| + 60% GHGE reduction | -64% | 98% | -46% | -100% | 63% | -4% | -26% | -44% | -31% | 29% | -10% |
| *GHGE minimised* | *-56%* | *58%* | *-100%* | *-100%* | *75%* | *-4%* | *-27%* | *-45%* | *-30%* | *31%* | *-19%* |

Table 3 Modelled dietary changes from the linear programming, Italy

|  | Fruits (g/day) | Vegetables (g/day) | Processed meat (g/day) | Red or livestock meat (g/day) | Fibre (g/day) | Sodium (g/day) | Total fat (% total energy) | Saturated fat (% total energy) | MUFA (% total energy) | PUFA (% total energy) | Dietary cholesterol (mg/d) |
| --- | --- | --- | --- | --- | --- | --- | --- | --- | --- | --- | --- |
| Men - baseline mean (SD) | 224 (157) | 161 (120) | 38 (22) | 65 (41) | 19 (6) | 2.1 (0.8) | 36% (5%) | 11% (2%) | 17% (3%) | 5% (1%) | 318 (125) |
| Diet meets recommendations | 69% | 65% | -71% | -29% | 32% | 14% | -11% | -10% | -11% | -13% | -9% |
| + no GHGE increase | 69% | 65% | -71% | -29% | 32% | 14% | -11% | -10% | -11% | -13% | -9% |
| + 10% GHGE reduction | 69% | 61% | -64% | -43% | 32% | 15% | -11% | -10% | -11% | -12% | -10% |
| + 20% GHGE reduction | 69% | 30% | -59% | -64% | 32% | 15% | -10% | -10% | -10% | -8% | -11% |
| + 30% GHGE reduction | 69% | 33% | -64% | -77% | 32% | 15% | -7% | -10% | -6% | -6% | -14% |
| + 40% GHGE reduction | 44% | 64% | -68% | -77% | 32% | 15% | -8% | -10% | -7% | -9% | -18% |
| + 50% GHGE reduction | 55% | 47% | -61% | -96% | 37% | 15% | -8% | -10% | -6% | -9% | -18% |
| + 60% GHGE reduction | 21% | 100% | -100% | -100% | 43% | 6% | -15% | -29% | -3% | -11% | -51% |
| *GHGE minimised* | *8%* | *113%* | *-100%* | *-100%* | *84%* | *15%* | *-8%* | *-28%* | *-15%* | *72%* | *-68%* |
| Women - baseline mean (SD) | 237 (146) | 152 (101) | 30 (15) | 56 (30) | 17 (6) | 1.6 (0.6) | 37% (5%) | 11% (2%) | 18% (3%) | 5% (1%) | 257 (102) |
| Diet meets recommendations | 70% | 81% | -83% | 52% | 45% | 16% | -12% | -12% | -12% | -12% | 4% |
| + no GHGE increase | 70% | 81% | -77% | -22% | 45% | 15% | -10% | -12% | -8% | -6% | 2% |
| + 10% GHGE reduction | 70% | 81% | -69% | -41% | 45% | 15% | -9% | -12% | -6% | -4% | 15% |
| + 20% GHGE reduction | 70% | 81% | -61% | -59% | 45% | 15% | -7% | -12% | -4% | -1% | 29% |
| + 30% GHGE reduction | 70% | 81% | -83% | -77% | 47% | 11% | -7% | -12% | -4% | -1% | 42% |
| + 40% GHGE reduction | 70% | 81% | -83% | -81% | 52% | 16% | -9% | -12% | -8% | -6% | 48% |
| + 50% GHGE reduction | 70% | 81% | -83% | -100% | 63% | 19% | -21% | -26% | -19% | -13% | 2% |
| + 60% GHGE reduction | 47% | 135% | -100% | -100% | 78% | 19% | -20% | -35% | -13% | -1% | -45% |
| *GHGE minimised* | *-4%* | *52%* | *-100%* | *-100%* | *78%* | *32%* | *-15%* | *-32%* | *-23%* | *52%* | *-65%* |

Table 4 Modelled dietary changes from the linear programming, Sweden

|  | Fruits (g/day) | Vegetables (g/day) | Processed meat (g/day) | Red or livestock meat (g/day) | Fibre (g/day) | Sodium (g/day) | Total fat (% total energy) | Saturated fat (% total energy) | MUFA (% total energy) | PUFA (% total energy) | Dietary cholesterol (mg/d) |
| --- | --- | --- | --- | --- | --- | --- | --- | --- | --- | --- | --- |
| Men - baseline mean (SD) | 371 (315) | 147 (111) | 81 (77) | 94 (60) | 21 (9) | 3.5 (1.2) | 37% (7%) | 14% (3%) | 13% (3%) | 6% (2%) | 294 (166) |
| Diet meets recommendations | 35% | 86% | -95% | 27% | 20% | -34% | -23% | -22% | -25% | -23% | 2% |
| + no GHGE increase | 35% | 68% | -94% | 0% | 24% | -34% | -27% | -22% | -30% | -37% | 5% |
| + 10% GHGE reduction | 35% | 45% | -95% | 0% | 26% | -34% | -27% | -22% | -30% | -38% | 18% |
| + 20% GHGE reduction | 35% | 41% | -94% | 16% | 28% | -34% | -27% | -22% | -31% | -37% | 16% |
| + 30% GHGE reduction | 35% | 37% | -95% | -42% | 40% | -34% | -21% | -22% | -23% | -21% | 7% |
| + 40% GHGE reduction | 35% | 36% | -95% | -40% | 43% | -34% | -19% | -22% | -20% | -16% | 7% |
| + 50% GHGE reduction | 61% | -13% | -93% | -78% | 54% | -34% | -20% | -22% | -21% | -18% | -4% |
| + 60% GHGE reduction | 15% | 33% | -95% | -100% | 80% | -34% | -21% | -29% | -19% | -9% | -14% |
| + 70% GHGE reduction | 30% | 9% | -88% | -100% | 100% | -34% | -12% | -25% | -16% | 20% | -24% |
| *GHGE minimised* | *42%* | *-50%* | *-91%* | *-100%* | *106%* | *-34%* | *-1%* | *-22%* | *-6%* | *62%* | *-74%* |
| Women - baseline mean (SD) | 338 (282) | 129 (94) | 49 (37) | 72 (42) | 20 (7) | 2.6 (0.8) | 37% (7%) | 14% (3%) | 13% (3%) | 6% (2%) | 211 (109) |
| Diet meets recommendations | 81% | 61% | -59% | 40% | 36% | -14% | -17% | -23% | -22% | 1% | 33% |
| + no GHGE increase | 61% | 61% | -59% | -41% | 39% | -14% | -18% | -23% | -24% | 2% | 31% |
| + 10% GHGE reduction | 56% | 61% | -59% | -63% | 40% | -14% | -17% | -23% | -23% | 8% | 30% |
| + 20% GHGE reduction | 67% | 61% | -59% | -72% | 45% | -14% | -16% | -23% | -23% | 13% | 23% |
| + 30% GHGE reduction | 60% | 61% | -59% | -83% | 45% | -14% | -14% | -23% | -20% | 17% | 22% |
| + 40% GHGE reduction | 9% | 61% | -59% | -99% | 51% | -14% | -8% | -23% | -10% | 28% | 14% |
| + 50% GHGE reduction | -28% | 61% | -59% | -100% | 59% | -14% | -9% | -23% | -11% | 26% | 3% |
| + 60% GHGE reduction | -30% | 25% | -59% | -100% | 75% | -14% | -8% | -23% | -11% | 37% | -42% |
| *GHGE minimised* | *12%* | *-23%* | *-59%* | *-100%* | *84%* | *-14%* | *-11%* | *-29%* | *-14%* | *35%* | *-60%* |

Table 5 Modelled dietary changes from the linear programming, Finland

|  | Fruits (g/day) | Vegetables (g/day) | Processed meat (g/day) | Red or livestock meat (g/day) | Fibre (g/day) | Sodium (g/day) | Total fat (% total energy) | Saturated fat (% total energy) | MUFA (% total energy) | PUFA (% total energy) | Dietary cholesterol (mg/d) |
| --- | --- | --- | --- | --- | --- | --- | --- | --- | --- | --- | --- |
| Men - baseline mean (SD) | 197 (146) | 185 (136) | 53 (37) | 62 (37) | 21 (8) | 3.7 (1.1) | 35% (5%) | 13% (2%) | 13% (2%) | 6% (2%) | 318 (130) |
| Diet meets recommendations | 13% | 90% | -98% | -79% | 32% | -31% | -19% | -29% | -23% | 3% | -38% |
| + no GHGE increase | 13% | 90% | -98% | -79% | 32% | -31% | -19% | -29% | -23% | 3% | -38% |
| + 10% GHGE reduction | 13% | 90% | -98% | -79% | 32% | -31% | -19% | -29% | -23% | 3% | -38% |
| + 20% GHGE reduction | 15% | 58% | -98% | -79% | 38% | -31% | -18% | -29% | -21% | 5% | -39% |
| + 30% GHGE reduction | 21% | -16% | -98% | -82% | 49% | -31% | -14% | -29% | -16% | 12% | -40% |
| + 40% GHGE reduction | 1% | -22% | -98% | -98% | 53% | -31% | -10% | -29% | -10% | 15% | -14% |
| + 50% GHGE reduction | -5% | -33% | -98% | -98% | 67% | -31% | -9% | -35% | -6% | 22% | -31% |
| + 60% GHGE reduction | -24% | -26% | -98% | -99% | 85% | -31% | -5% | -33% | -5% | 27% | -42% |
| + 70% GHGE reduction | -29% | -17% | -100% | -100% | 87% | -31% | -5% | -39% | -3% | 35% | -26% |
| *GHGE minimised* | *-40%* | *32%* | *-93%* | *-100%* | *99%* | *-31%* | *-5%* | *-51%* | *-2%* | *61%* | *-86%* |
| Women - baseline mean (SD) | 210 (132) | 162 (101) | 39 (23) | 49 (22) | 19 (6) | 2.8 (0.8) | 36% (5%) | 13% (3%) | 13% (2%) | 6% (1%) | 263 (111) |
| Diet meets recommendations | 48% | 29% | -96% | -18% | 67% | -7% | -26% | -47% | -29% | 10% | 5% |
| + no GHGE increase | -16% | 67% | -96% | -75% | 70% | -7% | -18% | -46% | -22% | 31% | 42% |
| + 10% GHGE reduction | -21% | 68% | -96% | -91% | 77% | -7% | -15% | -46% | -21% | 46% | 9% |
| + 20% GHGE reduction | -12% | 66% | -96% | -91% | 83% | -7% | -16% | -49% | -21% | 49% | 2% |
| + 30% GHGE reduction | 5% | 45% | -96% | -91% | 90% | -7% | -18% | -50% | -25% | 46% | 13% |
| + 40% GHGE reduction | -10% | -12% | -96% | -99% | 81% | -7% | -15% | -48% | -22% | 49% | 48% |
| + 50% GHGE reduction | -29% | -11% | -96% | -99% | 78% | -7% | -18% | -53% | -24% | 50% | 23% |
| + 60% GHGE reduction | -19% | -11% | -96% | -100% | 77% | -7% | -11% | -49% | -16% | 56% | 25% |
| + 70% GHGE reduction | -54% | 27% | -100% | -100% | 82% | -7% | -7% | -49% | -12% | 73% | -62% |
| *GHGE minimised* | *-33%* | *41%* | *-100%* | *-100%* | *103%* | *-21%* | *-11%* | *-47%* | *-14%* | *49%* | *-78%* |
