# Supplementary material for: Modelling the health co-benefits of sustainable diets in the UK, France, Finland, Italy and Sweden
Source: Eur J Clin Nutr. 2019 Feb 12;73(4):624–33. doi: 10.1038/s41430-019-0401-5 (PMC6484724; doi:10.1038/s41430-019-0401-5)
Supplement: Supplementary file 2 — Appendix 2 [file 41430_2019_401_MOESM2_ESM.docx]

**Appendix 2: Deaths averted by 2025 for all dietary scenarios in the United Kingdom, France, Finland, Italy and Sweden**

Figure 1 Deaths averted by 2025 in the UK

Figure 2 Deaths averted by 2025 in France

Figure 3 Deaths averted by 2025 in Italy

Figure 4 Deaths averted by 2025 in Sweden

Figure 5 Deaths averted by 2025 in Finland
