# Supplementary material for: Modelling the health co-benefits of sustainable diets in the UK, France, Finland, Italy and Sweden
Source: Eur J Clin Nutr. 2019 Feb 12;73(4):624–33. doi: 10.1038/s41430-019-0401-5 (PMC6484724; doi:10.1038/s41430-019-0401-5)
Supplement: Supplementary file 3 — Appendix 3 [file 41430_2019_401_MOESM3_ESM.docx]

**Appendix 3: Sensitivity of results for all dietary scenarios in the United Kingdom, France, Finland, Italy and Sweden**

Table 1 Sensitivity of the health gain (in millions of DALYs averted) to changes in time lags between dietary effects and disease outcomes, in the United Kingdom.

| **Dietary scenario** | **Men** | | | **Women** | | |
| --- | --- | --- | --- | --- | --- | --- |
|  | Base case* | No lags | Longer lags** | Base case* | No lags | Longer lags** |
| Diet meets nutrition recommendations | 15 | 16 (+6%) | 14 (-6%) | 13 | 13 (+7%) | 12 (-7%) |
| + no GHGE increase | 15 | 16 (+7%) | 14 (-6%) | 13 | 14 (+7%) | 12 (-7%) |
| + 10% GHGE reduction | 15 | 16 (+7%) | 14 (-7%) | 13 | 14 (+7%) | 12 (-7%) |
| + 20% GHGE reduction | 15 | 16 (+7%) | 14 (-6%) | 13 | 14 (+8%) | 12 (-7%) |
| + 30% GHGE reduction | 15 | 16 (+7%) | 14 (-6%) | 16 | 17 (+7%) | 15 (-7%) |
| + 40% GHGE reduction | 16 | 17 (+7%) | 15 (-7%) | 17 | 18 (+7%) | 16 (-7%) |
| + 50% GHGE reduction | 21 | 22 (+7%) | 19 (-7%) | 18 | 19 (+7%) | 16 (-7%) |
| + 60% GHGE reduction | 20 | 22 (+6%) | 19 (-6%) | 19 | 20 (+7%) | 18 (-7%) |
| + 70% GHGE reduction | 20 | 21 (+6%) | 18 (-6%) | — | — | — |
| GHGE minimised | 19 | 20 (+6%) | 18 (-6%) | 20 | 21 (+7%) | 18 (-7%) |

NB. GHGE – greenhouse gas emissions. * Base case lags of 5 years for CHD, stroke and diabetes, and 20 years for all cancers. * Longer lags of 10 years for CHD, stroke and diabetes, and 30 years for all cancers.

Table 2 Sensitivity of the health gain (in millions of DALYs averted) to disease trends, in the United Kingdom.

| **Dietary scenario** | **Men** |  | **Women** |  |
| --- | --- | --- | --- | --- |
|  | Base case* | No trends | Base case* | No trends |
| Diet meets nutrition recommendations | 15 | 14 (-6%) | 13 | 13 (+1%) |
| + no GHGE increase | 15 | 14 (-7%) | 13 | 13 (+1%) |
| + 10% GHGE reduction | 15 | 14 (-8%) | 13 | 13 (+2%) |
| + 20% GHGE reduction | 15 | 14 (-8%) | 13 | 13 (+2%) |
| + 30% GHGE reduction | 15 | 14 (-8%) | 16 | 16 (-1%) |
| + 40% GHGE reduction | 16 | 15 (-8%) | 17 | 17 (-1%) |
| + 50% GHGE reduction | 21 | 19 (-9%) | 18 | 18 (+0%) |
| + 60% GHGE reduction | 20 | 18 (-11%) | 19 | 19 (+1%) |
| + 70% GHGE reduction | 20 | 16 (-16%) | — | — |
| GHGE minimised | 19 | 16 (-18%) | 20 | 20 (+3%) |

NB. GHGE – greenhouse gas emissions. * Includes trends in mortality and YLD rates for all diseases.

Table 3 Sensitivity of the health gain (in millions of DALYs averted) to changes in time lags between dietary effects and disease outcomes, in France.

| **Dietary scenario** | **Men** | | | **Women** | | |
| --- | --- | --- | --- | --- | --- | --- |
|  | Base case* | No lags | Longer lags** | Base case* | No lags | Longer lags** |
| Diet meets nutrition recommendations | 7 | 7.8 (+11%) | 6.3 (-10%) | 7.9 | 8.6 (+10%) | 7.1 (-9%) |
| + no GHGE increase | 6.8 | 7.6 (+11%) | 6.1 (-10%) | 7.7 | 8.4 (+10%) | 7 (-9%) |
| + 10% GHGE reduction | 6.8 | 7.5 (+12%) | 6.1 (-10%) | 6.5 | 7 (+9%) | 5.9 (-9%) |
| + 20% GHGE reduction | 7 | 7.8 (+11%) | 6.3 (-10%) | 6.1 | 6.7 (+9%) | 5.6 (-9%) |
| + 30% GHGE reduction | 7.3 | 8.1 (+11%) | 6.5 (-10%) | 6.3 | 6.9 (+9%) | 5.7 (-9%) |
| + 40% GHGE reduction | 6.4 | 7.1 (+11%) | 5.8 (-10%) | 5.9 | 6.4 (+9%) | 5.3 (-9%) |
| + 50% GHGE reduction | 5.9 | 6.6 (+11%) | 5.4 (-10%) | 4.6 | 5.1 (+9%) | 4.2 (-9%) |
| + 60% GHGE reduction | 5.8 | 6.4 (+10%) | 5.3 (-9%) | 11 | 12 (+8%) | 10 (-8%) |
| + 70% GHGE reduction | 5.5 | 6.1 (+10%) | 5 (-9%) | 15 | 16 (+8%) | 14 (-8%) |
| GHGE minimised | 7 | 7.8 (+11%) | 6.3 (-10%) | 7.9 | 8.6 (+10%) | 7.1 (-9%) |

NB. GHGE – greenhouse gas emissions. * Base case lags of 5 years for CHD, stroke and diabetes, and 20 years for all cancers. * Longer lags of 10 years for CHD, stroke and diabetes, and 30 years for all cancers.

Table 4 Sensitivity of the health gain (in millions of DALYs averted) to disease trends, in France.

| **Dietary scenario** | **Men** |  | **Women** |  |
| --- | --- | --- | --- | --- |
|  | Base case* | No trends | Base case* | No trends |
| Diet meets nutrition recommendations | 7 | 8.7 (+24%) | 7.9 | 8.7 (+10%) |
| + no GHGE increase | 6.8 | 8.3 (+23%) | 7.7 | 8.4 (+10%) |
| + 10% GHGE reduction | 6.8 | 8.2 (+21%) | 6.5 | 7.4 (+14%) |
| + 20% GHGE reduction | 7 | 8.5 (+20%) | 6.1 | 7 (+14%) |
| + 30% GHGE reduction | 7.3 | 8.7 (+20%) | 6.3 | 7.2 (+14%) |
| + 40% GHGE reduction | 6.4 | 7.6 (+19%) | 5.9 | 6.7 (+15%) |
| + 50% GHGE reduction | 5.9 | 7 (+18%) | 4.6 | 5.4 (+16%) |
| + 60% GHGE reduction | 5.8 | 6.9 (+18%) | 11 | 12 (+6%) |
| + 70% GHGE reduction | 5.5 | 6.6 (+19%) | 15 | 16 (+4%) |
| GHGE minimised | 7 | 8.7 (+24%) | 7.9 | 8.7 (+10%) |

NB. GHGE – greenhouse gas emissions. * Includes trends in mortality and YLD rates for all diseases.

Table 5 Sensitivity of the health gain (in millions of DALYs averted) to changes in time lags between dietary effects and disease outcomes, in Italy.

| **Dietary scenario** | **Men** | | | **Women** | | |
| --- | --- | --- | --- | --- | --- | --- |
|  | Base case* | No lags | Longer lags** | Base case* | No lags | Longer lags** |
| Diet meets nutrition recommendations | 4.7 | 5.3 (+14%) | 4.2 (-11%) | 4.9 | 5.4 (+12%) | 4.4 (-10%) |
| + no GHGE increase | 4.7 | 5.3 (+14%) | 4.2 (-11%) | 5.1 | 5.7 (+11%) | 4.6 (-10%) |
| + 10% GHGE reduction | 4.6 | 5.3 (+14%) | 4.1 (-11%) | 5 | 5.5 (+11%) | 4.5 (-10%) |
| + 20% GHGE reduction | 4.2 | 4.8 (+14%) | 3.7 (-11%) | 4.8 | 5.4 (+11%) | 4.3 (-10%) |
| + 30% GHGE reduction | 4.5 | 5.1 (+14%) | 4 (-11%) | 5.5 | 6.2 (+13%) | 4.9 (-11%) |
| + 40% GHGE reduction | 4.6 | 5.3 (+14%) | 4.1 (-11%) | 5.7 | 6.4 (+14%) | 5 (-11%) |
| + 50% GHGE reduction | 7.4 | 8.6 (+16%) | 6.6 (-12%) | 11 | 13 (+11%) | 10 (-10%) |
| + 60% GHGE reduction | 12 | 14 (+13%) | 11 (-11%) | 15 | 16 (+11%) | 13 (-9%) |
| + 70% GHGE reduction | 13 | 14 (+13%) | 11 (-11%) | 13 | 14 (+11%) | 11 (-9%) |
| GHGE minimised | 4.7 | 5.3 (+14%) | 4.2 (-11%) | 4.9 | 5.4 (+12%) | 4.4 (-10%) |

NB. GHGE – greenhouse gas emissions. * Base case lags of 5 years for CHD, stroke and diabetes, and 20 years for all cancers. * Longer lags of 10 years for CHD, stroke and diabetes, and 30 years for all cancers.

Table 6 Sensitivity of the health gain (in millions of DALYs averted) to disease trends, in Italy.

| **Dietary scenario** | **Men** |  | **Women** |  |
| --- | --- | --- | --- | --- |
|  | Base case* | No trends | Base case* | No trends |
| Diet meets nutrition recommendations | 4.7 | 7.5 (+61%) | 4.9 | 7.1 (+46%) |
| + no GHGE increase | 4.7 | 7.6 (+61%) | 5.1 | 7.4 (+45%) |
| + 10% GHGE reduction | 4.6 | 7.4 (+61%) | 5 | 7.2 (+45%) |
| + 20% GHGE reduction | 4.2 | 6.7 (+58%) | 4.8 | 7 (+45%) |
| + 30% GHGE reduction | 4.5 | 7 (+57%) | 5.5 | 7.9 (+44%) |
| + 40% GHGE reduction | 4.6 | 7.3 (+57%) | 5.7 | 8.1 (+44%) |
| + 50% GHGE reduction | 7.4 | 11 (+48%) | 11 | 16 (+39%) |
| + 60% GHGE reduction | 12 | 18 (+42%) | 15 | 20 (+38%) |
| + 70% GHGE reduction | 13 | 18 (+43%) | 13 | 17 (+37%) |
| GHGE minimised | 4.7 | 7.5 (+61%) | 4.9 | 7.1 (+46%) |

NB. GHGE – greenhouse gas emissions. * Includes trends in mortality and YLD rates for all diseases.

Table 7 Sensitivity of the health gain (in millions of DALYs averted) to changes in time lags between dietary effects and disease outcomes, in Sweden.

| **Dietary scenario** | **Men** | | | **Women** | | |
| --- | --- | --- | --- | --- | --- | --- |
|  | Base case* | No lags | Longer lags** | Base case* | No lags | Longer lags** |
| Diet meets nutrition recommendations | 1.4 | 1.5 (+10%) | 1.3 (-10%) | 0.76 | 0.82 (+9%) | 0.69 (-9%) |
| + no GHGE increase | 1.3 | 1.4 (+11%) | 1.2 (-10%) | 0.8 | 0.87 (+9%) | 0.72 (-10%) |
| + 10% GHGE reduction | 1.3 | 1.4 (+11%) | 1.2 (-10%) | 0.79 | 0.86 (+9%) | 0.72 (-10%) |
| + 20% GHGE reduction | 1.2 | 1.3 (+11%) | 1.1 (-10%) | 0.84 | 0.92 (+9%) | 0.76 (-10%) |
| + 30% GHGE reduction | 1.3 | 1.5 (+11%) | 1.2 (-10%) | 0.86 | 0.95 (+10%) | 0.77 (-10%) |
| + 40% GHGE reduction | 1.3 | 1.4 (+11%) | 1.2 (-10%) | 1.5 | 1.6 (+10%) | 1.3 (-10%) |
| + 50% GHGE reduction | 1.1 | 1.3 (+12%) | 1 (-10%) | 1.5 | 1.6 (+9%) | 1.3 (-9%) |
| + 60% GHGE reduction | 2 | 2.2 (+10%) | 1.8 (-10%) | 1.4 | 1.6 (+9%) | 1.3 (-9%) |
| + 70% GHGE reduction | 1.8 | 2 (+10%) | 1.6 (-10%) | — | — | — |
| GHGE minimised | 1.8 | 2 (+9%) | 1.6 (-9%) | 1.5 | 1.6 (+9%) | 1.4 (-9%) |

NB. GHGE – greenhouse gas emissions. * Base case lags of 5 years for CHD, stroke and diabetes, and 20 years for all cancers. * Longer lags of 10 years for CHD, stroke and diabetes, and 30 years for all cancers.

Table 8 Sensitivity of the health gain (in millions of DALYs averted) to disease trends, in Sweden.

| **Dietary scenario** | **Men** |  | **Women** |  |
| --- | --- | --- | --- | --- |
|  | Base case* | No trends | Base case* | No trends |
| Diet meets nutrition recommendations | 1.4 | 1.7 (+23%) | 0.76 | 1 (+33%) |
| + no GHGE increase | 1.3 | 1.6 (+22%) | 0.8 | 1 (+29%) |
| + 10% GHGE reduction | 1.3 | 1.5 (+16%) | 0.79 | 1 (+29%) |
| + 20% GHGE reduction | 1.2 | 1.4 (+18%) | 0.84 | 1.1 (+30%) |
| + 30% GHGE reduction | 1.3 | 1.5 (+17%) | 0.86 | 1.1 (+29%) |
| + 40% GHGE reduction | 1.3 | 1.5 (+17%) | 1.5 | 1.7 (+13%) |
| + 50% GHGE reduction | 1.1 | 1.3 (+15%) | 1.5 | 1.6 (+10%) |
| + 60% GHGE reduction | 2 | 2.2 (+12%) | 1.4 | 1.6 (+10%) |
| + 70% GHGE reduction | 1.8 | 2.1 (+14%) | — | — |
| GHGE minimised | 1.8 | 2 (+11%) | 1.5 | 1.7 (+11%) |

NB. GHGE – greenhouse gas emissions. * Includes trends in mortality and YLD rates for all diseases.

Table 9 Sensitivity of the health gain (in millions of DALYs averted) to changes in time lags between dietary effects and disease outcomes, in Finland.

| **Dietary scenario** | **Men** | | | **Women** | | |
| --- | --- | --- | --- | --- | --- | --- |
|  | Base case* | No lags | Longer lags** | Base case* | No lags | Longer lags** |
| Diet meets nutrition recommendations | 2.3 | 2.5 (+7%) | 2.1 (-7%) | 1.5 | 1.7 (+8%) | 1.4 (-8%) |
| + no GHGE increase | 2.3 | 2.5 (+7%) | 2.1 (-7%) | 1.5 | 1.6 (+9%) | 1.4 (-8%) |
| + 10% GHGE reduction | 2.3 | 2.5 (+7%) | 2.1 (-7%) | 1.7 | 1.8 (+9%) | 1.5 (-8%) |
| + 20% GHGE reduction | 2.3 | 2.4 (+7%) | 2.1 (-7%) | 1.7 | 1.9 (+9%) | 1.6 (-8%) |
| + 30% GHGE reduction | 2.2 | 2.3 (+7%) | 2 (-7%) | 1.8 | 1.9 (+9%) | 1.6 (-8%) |
| + 40% GHGE reduction | 2.5 | 2.7 (+7%) | 2.3 (-7%) | 1.9 | 2.1 (+9%) | 1.7 (-8%) |
| + 50% GHGE reduction | 2.5 | 2.6 (+7%) | 2.3 (-7%) | 1.8 | 2 (+8%) | 1.7 (-8%) |
| + 60% GHGE reduction | 2.5 | 2.7 (+7%) | 2.3 (-7%) | 1.9 | 2.1 (+8%) | 1.8 (-8%) |
| + 70% GHGE reduction | 2.6 | 2.8 (+6%) | 2.4 (-7%) | 2.1 | 2.2 (+8%) | 1.9 (-7%) |
| GHGE minimised | 2.5 | 2.7 (+7%) | 2.3 (-7%) | 2.3 | 2.5 (+8%) | 2.1 (-8%) |

NB. GHGE – greenhouse gas emissions. * Base case lags of 5 years for CHD, stroke and diabetes, and 20 years for all cancers. * Longer lags of 10 years for CHD, stroke and diabetes, and 30 years for all cancers.

Table 10 Sensitivity of the health gain (in millions of DALYs averted) to disease trends, in Finland.

| **Dietary scenario** | **Men** |  | **Women** |  |
| --- | --- | --- | --- | --- |
|  | Base case* | No trends | Base case* | No trends |
| Diet meets nutrition recommendations | 2.3 | 2.5 (+7%) | 1.5 | 1.7 (+11%) |
| + no GHGE increase | 2.3 | 2.5 (+7%) | 1.5 | 1.6 (+6%) |
| + 10% GHGE reduction | 2.3 | 2.5 (+7%) | 1.7 | 1.8 (+7%) |
| + 20% GHGE reduction | 2.3 | 2.4 (+4%) | 1.7 | 1.9 (+8%) |
| + 30% GHGE reduction | 2.2 | 2.1 (-3%) | 1.8 | 1.9 (+9%) |
| + 40% GHGE reduction | 2.5 | 2.2 (-10%) | 1.9 | 1.9 (+1%) |
| + 50% GHGE reduction | 2.5 | 2.2 (-10%) | 1.8 | 1.8 (-1%) |
| + 60% GHGE reduction | 2.5 | 2.3 (-10%) | 1.9 | 2 (+1%) |
| + 70% GHGE reduction | 2.6 | 2.3 (-11%) | 2.1 | 2.1 (+1%) |
| GHGE minimised | 2.5 | 2.5 (-0.1%) | 2.3 | 2.5 (+8%) |

NB. GHGE – greenhouse gas emissions. * Includes trends in mortality and YLD rates for all diseases.
